# Influence of radiotherapy interruption on esophageal cancer with intensity-modulated radiotherapy: a retrospective study

**DOI:** 10.1186/s12885-024-12383-7

**Published:** 2024-05-27

**Authors:** Yanhong Mou, Peng Liang, Xun Cheng, Xin He, Jun Zhang, Liangzhong Liu, Qiang Liu

**Affiliations:** 1 Physics and Technique Department of Radiation Oncology, Chongqing University Three Gorges Hospital, Wanzhou, Chongqing 40400 People’s Republic of China; 2 Department of Oncology, Chongqing University Three Gorges Hospital, Wanzhou, Chongqing 404000 People’s Republic of China

**Keywords:** Esophageal cancer, Radiotherapy, Radiotherapy interruption, Delayed time

## Abstract

**Background:**

Radiotherapy interruption (RTI) prolongs the overall total treatment time and leads to local control loss in many cancers, but it is unclear in esophageal cancer. We aimed to evaluate the influence of RTI on the overall survival (OS), progression-free survival (PFS), and local-regional recurrence-free survival (LRFS) of patients with esophageal cancer undergoing chemoradiotherapy.

**Methods:**

A total of 299 patients with esophageal squamous cell carcinoma from 2017 to 2019 were retrospectively analyzed to investigate the effect of RTI on OS, PFS, and LRFS. The delayed time of radiotherapy interruption was calculated as the actual radiation treatment time minus the scheduled time. The univariate and multivariate analyses were performed by the COX proportional hazards regression models, and the survival analysis was performed through the Kaplan‒Meier method, and compared with the log-rank test.

**Results:**

The 3-year OS, PFS, and LRFS rates were 53.0%, 42.0%, and 48.0%, respectively. The univariate and multivariate analyses showed that the delayed time > 3 days was an independent adverse prognostic factor for OS (HR = 1.68, 95% CI 1.10–2.55, *p* = 0.016), and LRFS (HR = 1.74, 95% CI 1.18–2.57, *p* = 0.006). The patient with a delayed time of > 3 days had poorer survival rates of OS, and LRFS than patients with a delayed time of ≤ 3 days (OS, *p* = 0.047; LRFS, *p* = 0.013), and the survival outcomes of patients with shorter delayed time (1–3 days) were slightly different from the patients without interruptions. The impact of delay time on PFS is not statistically significant, but the survival outcomes of the two groups were slightly different.

**Conclusion:**

There was a significant correlation between delayed time and local control of esophageal cancer. The delayed time for more than 3 days might decrease the survival outcome, and increase the local recurrence risk.

**Supplementary Information:**

The online version contains supplementary material available at 10.1186/s12885-024-12383-7.

## Background

Esophageal cancer, a prominent gastrointestinal cancer, has a poor prognosis. The 3-year overall survival (OS) was approximately 40% in the ARTDECO study, for patients who had undergone chemoradiotherapy [1]. It has been confirmed that the prognostic factors for esophageal cancer are numerous and complex based on previous studies, such as, body mass index(BMI) [2], smoking [3], gross tumor volume [4], and positive lymph nodes [5].

Radiotherapy (RT), as one of the common treatment options, undergoes unscheduled treatment when radiotherapy interruption (RTI) occurs. RTI is affected by equipment breakdown, public holidays, and toxicity [6], prolongs the overall treatment time, and significantly affects local tumor control. The severity is determined by the delayed time. Some studies have shown that the five-year OS decreased by 15% with more than 5 days of RTI for nasopharyngeal cancer using intensity-modulated radiotherapy [7], and only one day of RTI increased the risk of local relapse by 4.8% and led to a decrease in the local tumor control by 1.4% for laryngeal cancer [8]. The existing data about RTI were published on head and neck cancer, cervical cancer, prostate cancer, etc., but its use is not clear for esophageal cancer. We aim to investigate the influence of RTI on the survival outcome of patients with esophageal cancer.

## Methods

### Patients

A total of 299 patients with esophageal squamous cell carcinoma (ESCC) were selected between 2017 and 2019. All the patients newly received chemoradiotherapy (including concurrent chemoradiotherapy and induction chemotherapy). A total of 228 patients received intensity-modulated radiation therapy (IMRT), while the others underwent volume-modulated arc radiotherapy (VMAT). The characteristics are summarized in Table 1.

**Table 1:** Characteristics of 299 patients

Characteristic	Patients No.(%)
*Gender*	
Male	219(73.24%)
Female	80(26.76%)
*Age, years*	299
Median(range)	67(46–87)
*Tumor site*	
Upper third	117(39.13%)
Middle third	156 (52.17%)
Lower third	26(8.70%)
*Drinking*	
No	146(48.83%)
Yes	153(51.17%)
*Smoking*	
No	139(46.49%)
Yes	160 (53.51%)
*Diabetes*	
No	290(96.99%)
Yes	9(3.01%)
*Hypertension*	
No	254(84.95%)
Yes	45(15.05%)
*KPS*	
70	3
80	249
90	47
*Esophageal fistula*	
No	274(91.64%)
Yes	25(8.36%)
*Chemotherapy*	
Paclitaxel and platinum	157(52.51%)
Platinum and fluorouracil	10(3.34%)
Platinum and tegeo	30(10.03%)
Tegeo	69(23.08%)
Others	33(11.04%)
*Radiotherapy*	
IMRT	228(76.25%)
VMAT	71(23.75%)
*BMI*	
Underweight (< 18.5 *kg/m*^*2*^)	21(7.02%)
Nomalweight (18.5 to 24 *kg/m*^*2*^)	222(74.25%)
Overweight (≥ 24 *kg/m*^*2*^)	56(18.73%)
*T stage*	
1–2	103(34.45%)
3	143(47.83%)
4	53(17.72%)
*N stage*	
0	37(12.37%)
1	106(35.45%)
2	130(43.48%)
3	26(8.70%)
*GTV, cc*	299
Median(range)	30.21(3.55-250.29)
*GTVnd, cc*	299
Median(range)	3.86(0-116.45)
*Delayed time, days*	147(49.16%)
Median(range)	3 (1–18)

†BMI was stratified into three categories (only 6 cases for obesity, BMI ≥ 28 kg/m^2^)

### Radiotherapy

The planning CT was conducted based on the CT simulation planning system, with an axial slice thickness of 5 mm, ranging from the bottom of the mandible to 5 cm below the costophrenic angle, including the whole neck and lung. The primary gross tumor (GTV) and positive lymph nodes (GTVnd) were delineated on CT-enhanced images, combined with barium meal X-ray and endoscopic ultrasound. The clinical tumor volume (CTV) of the primary gross tumor was outlined by the expansion of the GTV (5 mm cm top-bottom, 5–6 mm left-right, 5–6 mm anterior-posterior) and the clinical tumor volume (CTVnd) of the positive lymph nodes referred to the latest UICC/AJCC guidelines. The planning tumor volume (PTV) was expanded from 5 mm of CTV and CTVnd. The IMRT plan was designed with 5–7 beams from the patient’s anterior and posterior sides to effectively reduce the lung dosage. The VMAT plan was designed with 2 arcs (330º-180º for CW, 180º-330º for CCW). The limits of OARs were as follows: heart V30 ≤ 40%, V40 ≤ 30%; lungs V5 ≤ 65%, V20 ≤ 30%, V30 ≤ 18%; mean lung dose ≤ 17 Gy; and spinal cord maximum dose ≤ 45 Gy. The dose distribution was calculated with Philips Radiation Oncology’s Pinnacle^3^ collapsed cone convolution superposition (CCCS) with 58.80 Gy to 66 Gy for GTV/GTVnd and 48.60 Gy to 60 Gy for PTV in 27 to 30 fractions scheduled once a day for 5 days per week. The delayed time was calculated as the actual radiation treatment time minus the scheduled time.

### Follow-up and endpoint

Follow-up was performed with barium meal X-ray examination, endoscopic ultrasound, or CT scan every 3 months, 6 months, and 1 year for the first year, second year, and thereafter.

Overall survival refers to the event from the beginning of treatment to death (for any reason) or the last follow-up, and the endpoint is death. Progression-free survival (PFS) refers to the time from the beginning of treatment to tumor progression (in any aspect) or death (for any reason); the endpoint is progression or death. Local-regional recurrence-free survival (LRFS) refers to the time from the beginning of treatment to the date of local-regional recurrence or death (for any reason); the endpoint is recurrence or death.

### Data Collection and Analysis

The data were collected and transformed into categorical variables by reference [9], including BMI, smoking history, alcohol consumption, TNM stage, GTV, GTVnd, and delayed time. The cutoff value was calculated by the survival package of the R project, The survival curves were drawn using Kaplan‒Meier methods and compared with the log-rank test. The univariate and multivariate analysis was performed by the Cox proportional hazards regression model to calculate the hazard ratio (HR) with 95% confidence intervals (CIs). A *p*-value of < 0.05 was statistically significant.

## Results

### RT interruption events

**Fig. 1:**
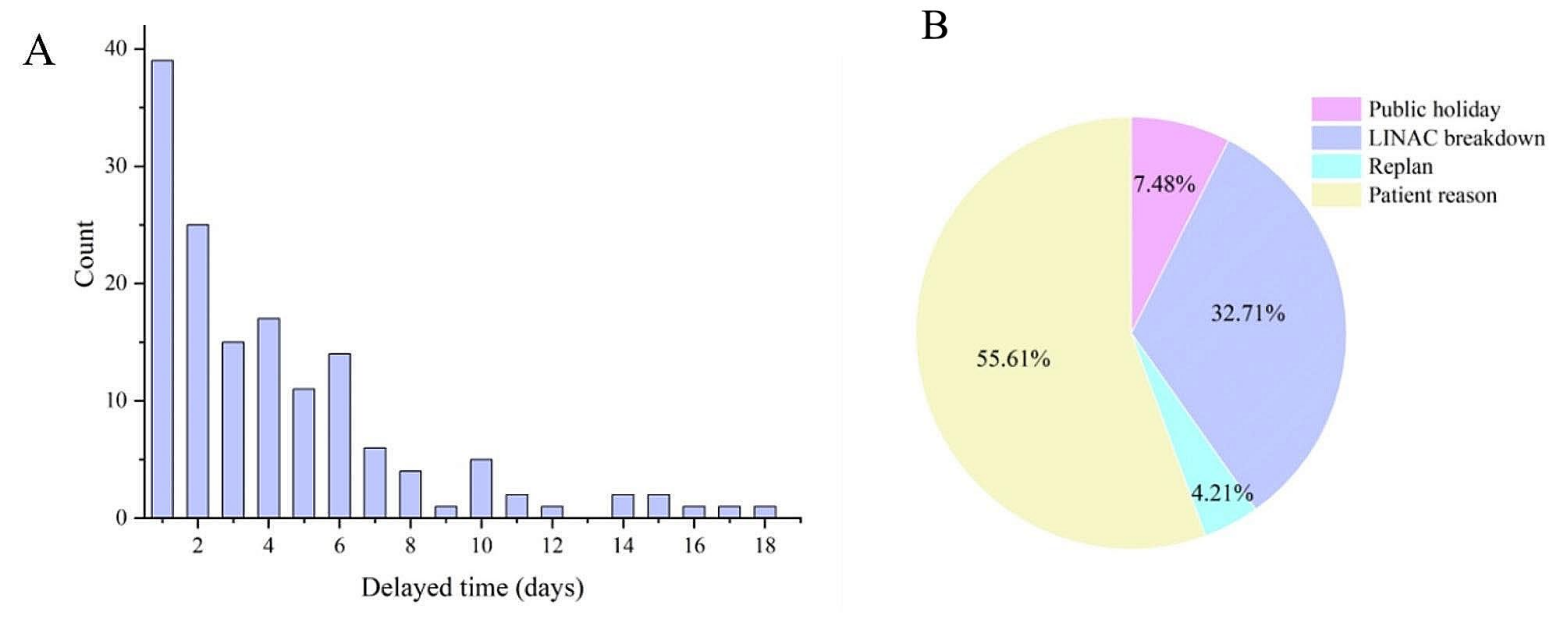
Details of radiotherapy interruption. (**A**), the bar chart of the delayed time, the counts were calculated by the number of patients; (**B**) the pie chart of the interruption’s reason, the counts were calculated based on the interruptions

A total of 147 patients suffered RT treatment interruption, and 57 patients had multiple interruptions. There were 70 interruptions of linear accelerator (LINAC) breakdown, 9 interruptions of replans, 16 interruptions of public holidays, and the rest were patient reasons such as toxicity and complications, as shown in Fig. 1.

### Cutoff

The cutpoints were calculated by the survival package of the R project. The cutpoints of Age, GTV, and GTVnd were 74 years, 23.27 cc, and 8.50 cc respectively. The cutoff of the delayed time was 3 days, The patients were divided into the delayed time > 3 days group and the delayed time ≤ 3 days group. The patients’ characteristics were compared with different delayed time groups using Fisher’s precision probability test, summarized in Table 2.

**Table 2:** Comparison of patients’ characteristics between delayed time ≤ 3 days and > 3 days

Characteristics	Patients No.(%)		*p*
	delayed time ≤ 3 days (*n* = 231)	delayed time > 3 days (*n* = 68)	
*Gender*			
Female	61(26.41%)	19(27.94%)	0.876
Male	170(73.59%)	49(72.06%)	
*Age, years*			
≤ 74	201(87.01%)	63(92.65%)	0.283
> 74	30(12.99%)	5(7.35%)	
*Drinking*			
No	108(46.75%)	38(55.88%)	0.215
Yes	123(53.25%)	30(44.12%)	
*Smoking*			
No	104(45.02%)	35(51.47%)	0.407
Yes	127(54.98%)	33(48.53%)	
*Diabetes*			
No	223(96.54%)	67(98.53%)	0.689
Yes	8(3.45%)	1(1.47%)	
*Hypertension*			
No	202(87.45%)	52(76.47%)	0.034
Yes	29(12.55%)	16(23.53%)	
*BMI, kg/m* ^*2*^			
< 18.5	17(7.36%)	4(5.88%)	0.886
18.5–24	172(74.46%)	50(73.53%)	
≥ 24	42(18.18%)	14(20.59%)	
*KPS*			
70	2(0.09%)	1(1.47%)	0.081
80	198(85.71%)	51(75.00%)	
90	31(13.42%)	16(23.53%)	
*T category*			
1–2	78(33.77%)	25(36.76%)	0.591
3	114(49.35%)	29(42.65%)	
4	39(16.88%)	14(20.59%)	
*N category*			
0	28(12.12%)	9(13.24%)	0.876
1	84(36.36%)	22(32.35%)	
2	100(43.29%)	30(44.12%)	
3	19(8.23%)	7(10.29%)	
*GTV, cc*			
≤ 23.27	80(34.63%)	23(33.82%)	1.000
> 23.27	151(65.37%)	45(66.18%)	
*GTVnd, cc*			
≤ 8.50	169(73.16%)	41(60.29%)	0.05
> 8.50	62(26.84%)	27(39.71%)	

#### Survival

The 3-year OS, PFS, and LRFS rates were 53.0%, 42.0%, and 48.0%, respectively, with a follow-up range of 2–62 months. The median OS time was 14 months (range 2–50 months) for 132 death events.

### Univariate and multivariate analysis

**Fig. 2:**
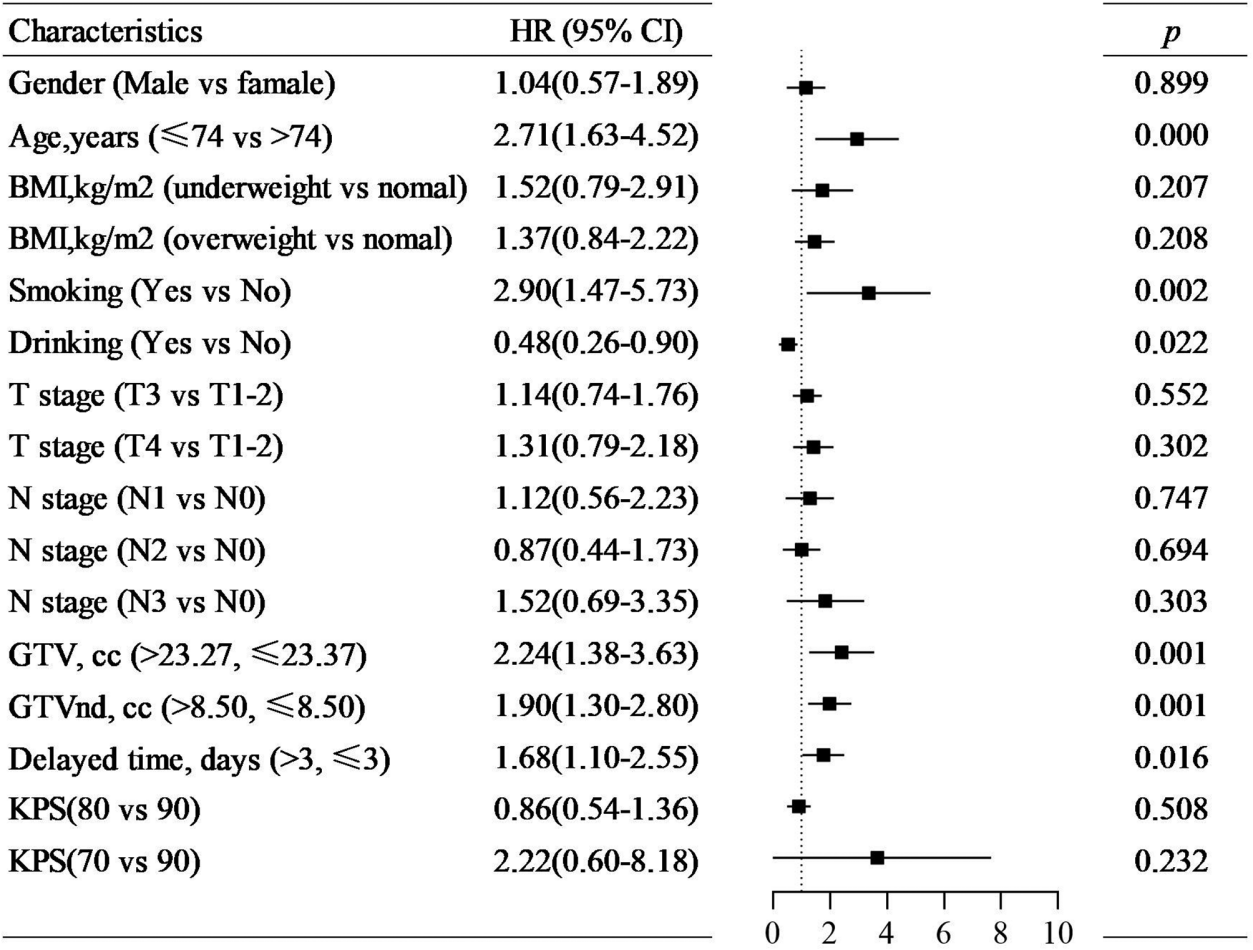
Multivariable analysis of Cox proportional hazards regression model to estimate the risk of overall survival

**Fig. 3:**
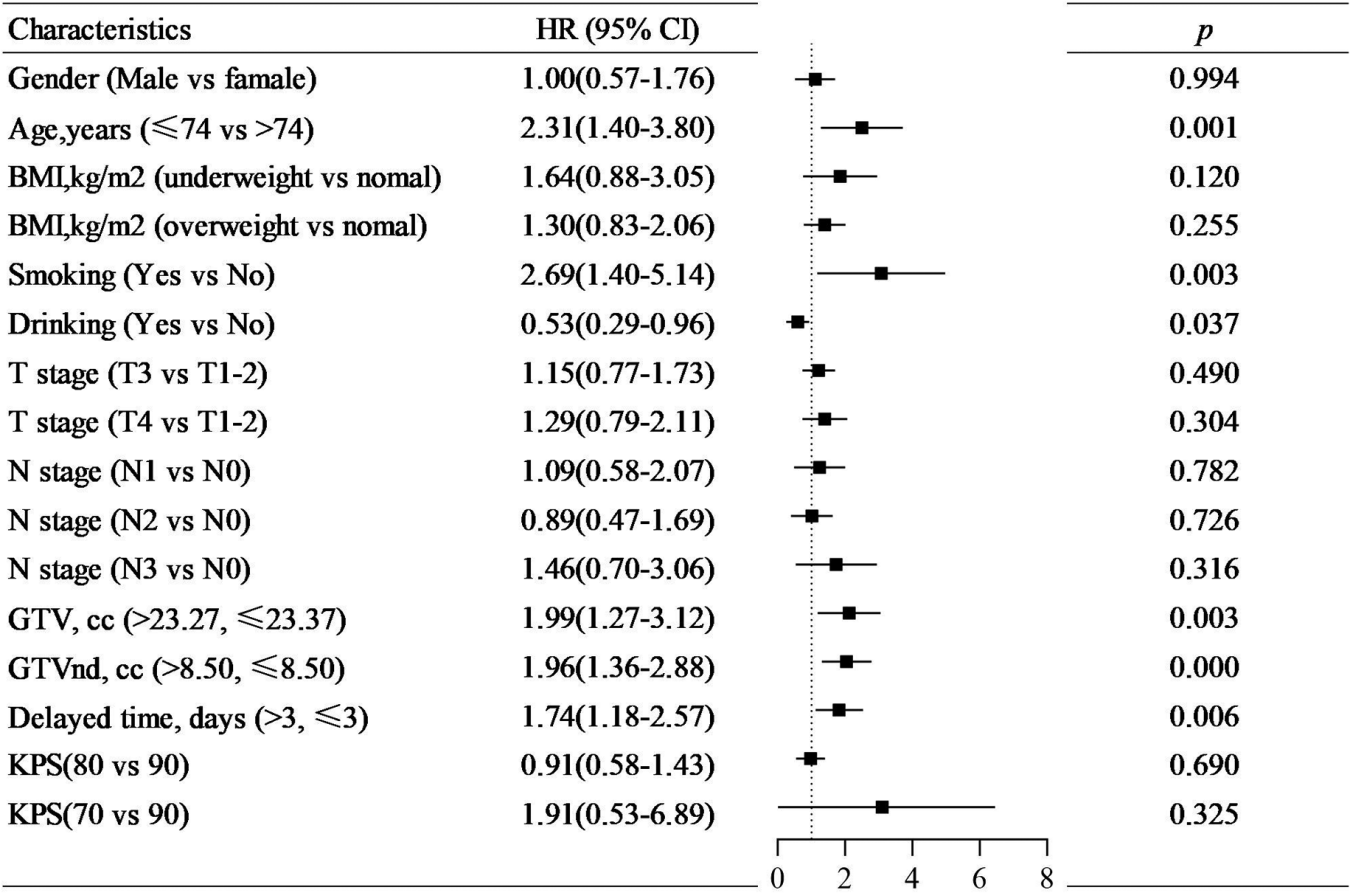
Multivariable analysis of Cox proportional hazards regression model to estimate the risk of local-regional recurrence-free survival

**Fig. 4:**
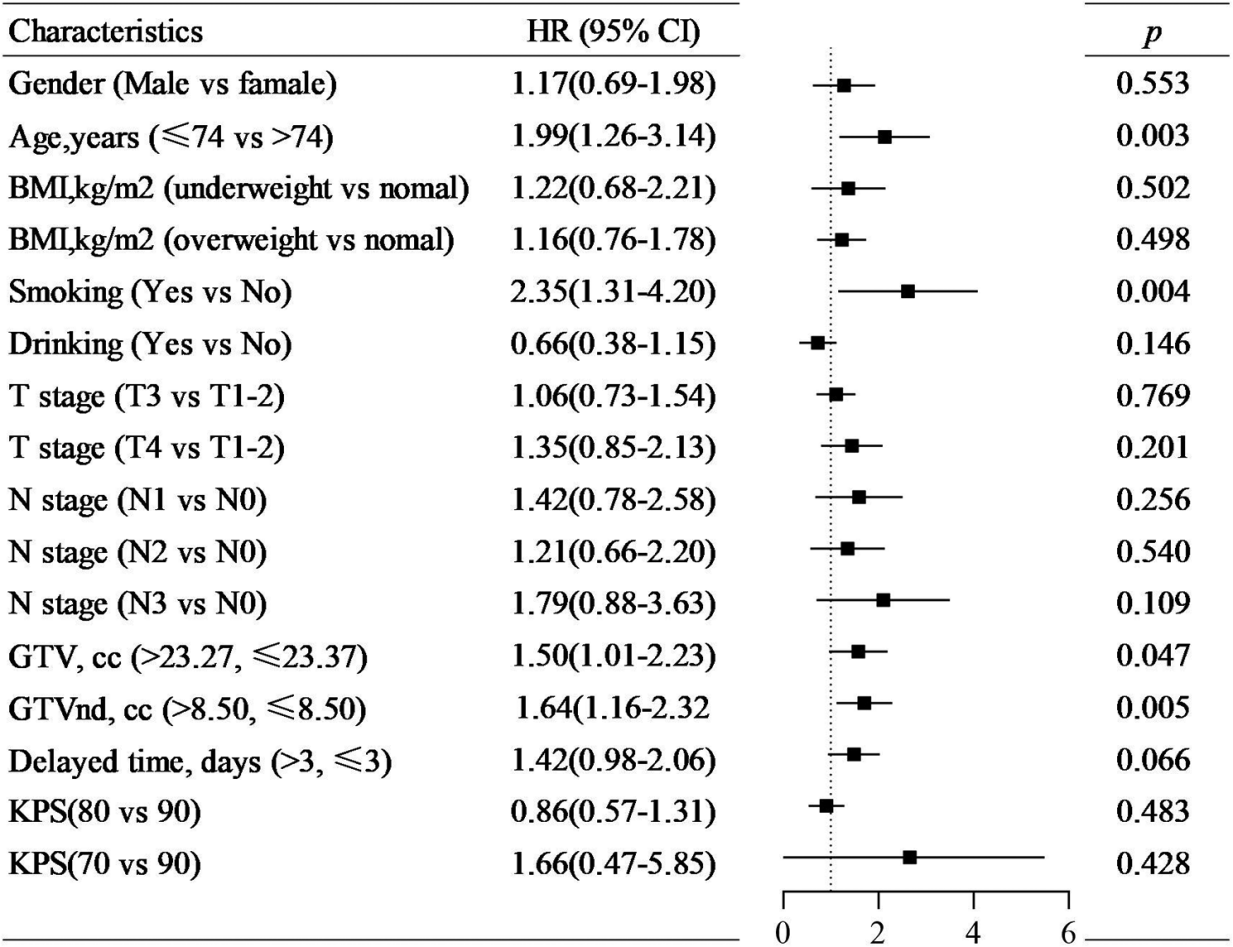
Multivariable analysis of Cox proportional hazards regression model to estimate the risk of progression-free survival

**Fig. 5:**
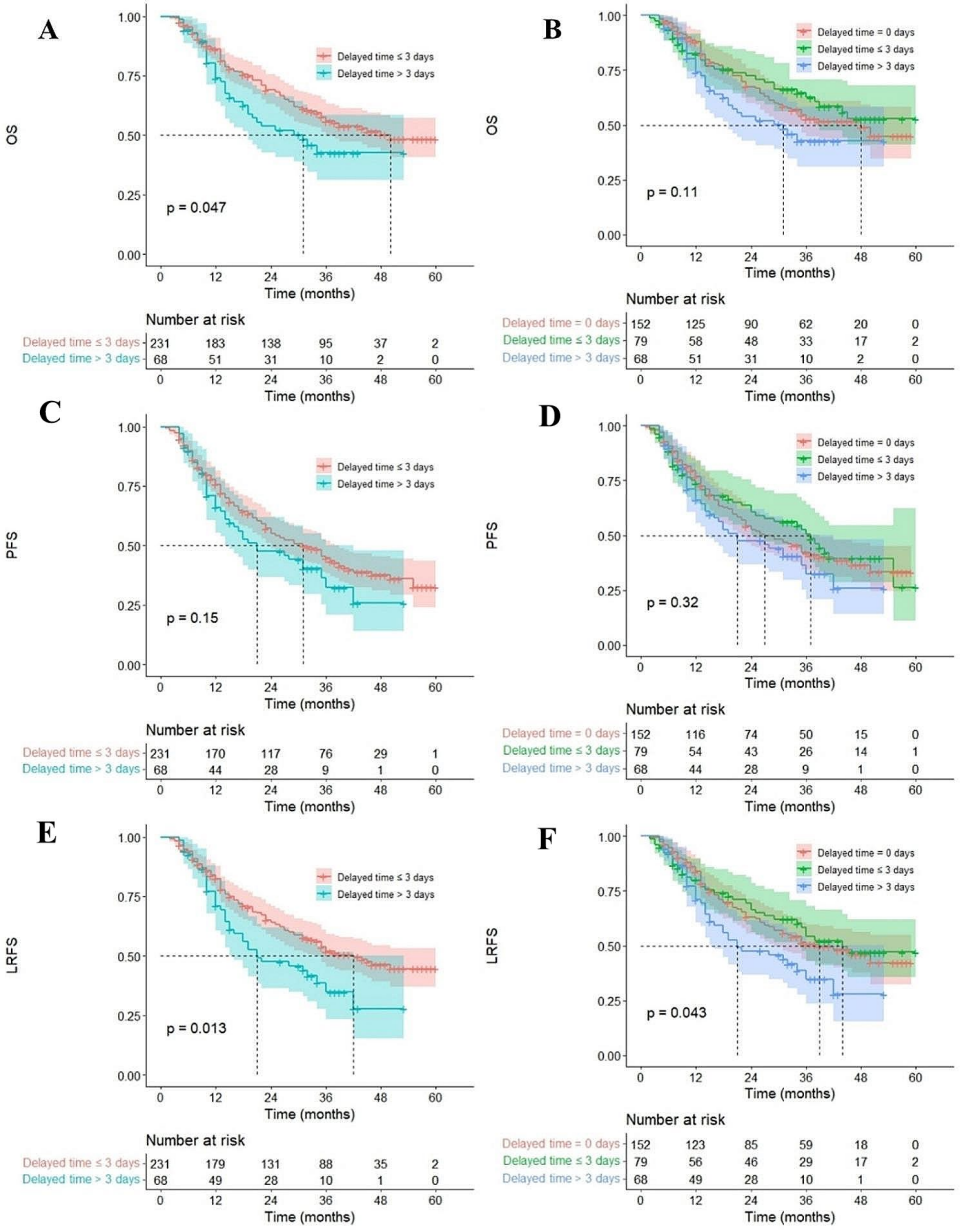
Kaplan-Meier representation of survival outcomes. *OS* overall survival, *PFS* Progression-free survival, *LRFS* Local-regional recurrence-free survival. (**A**, **C**, and **E**, the patients with a delayed time ≤ 3 days group included non-interruption), (**B**, **D**, and **F**, the patients with a delayed time ≤ 3 days excluded non-interruption)

It was shown that age, gender, smoking, T category, N category, GTV, and GTVnd were significantly different in OS, PFS, and LRFS of patients by the univariate analysis listed in Table 3 (*p* < 0.05). The delayed time had a significant impact on LRFS. From the multivariate analysis result, age, smoking, delayed time, GTV, and GTVnd were independent prognostic factors of OS and LRFS (*p* < 0.05, Fig. 2 and Fig. 3). While age, smoking, GTV, and GTVnd were independent prognostic factors of PFS (*p* < 0.05, Fig. 4). The mortality risk of patients with a delayed time > 3 days was 1.68 (95% CI 1.10–2.55, *p* = 0.016) times higher than those of patients with a delayed time ≤ 3 days. The local regional recurrence risk of patients with a delayed time > 3 days was 1.74 (95% CI 1.18–2.57, *p* = 0.006) times higher than those of patients with a delayed time ≤ 3 days. From the Kaplan‒Meier curves, the OS and LRFS outcomes of delayed time > 3 days were poorer than those of patients with delayed time ≤ 3 days (*p* < 0.05, Fig. 5). Furthermore, the delayed time was divided into non-interruptions (0 days), shorter delayed time (1 to 3 days), and longer delayed time (> 3 days), it was found that the outcomes of patients with shorter delayed were slightly different from the non-interruptions.

**Table 3:** Univariate analysis for the survival outcome

Characteristics	No. of	OS		PFS		LRFS	
	patient	HR (95%CI)	*p*	HR (95%CI)	*p*	HR (95%CI)	*p*
*Gender*							
Female	80	Reference		Reference		Reference	
Male	219	1.52 (1.01–2.32)	0.046	1.67 (1.17–2.46)	0.005	1.49(1.01–2.20)	0.043
*Age, years*							
≤ 74	272	Reference		Reference		Reference	
> 74	27	2.01 (1.27–3.18)	0.003	1.85(1.21–2.84)	0.005	1.74(1.11–2.74)	0.017
*Drinking*							
No	146	Reference		Reference		Reference	
Yes	153	1.21 (0.86–1.71)	0.275	1.43 (1.05–1.94)	0.022	1.22(0.88–1.70)	0.225
*Smoking*							
No	139	Reference		Reference		Reference	
Yes	160	1.62(1.14–2.31)	0.007	1.80 (1.32–2.46)	< 0.001	1.62(1.16–2.26)	0.005
*BMI, kg/m* ^*2*^							
< 18.5	21	1.64(0.88–3.06)	0.122	1.43(0.81–2.54)	0.218	1.73(0.95–3.16)	0.071
18.5–24	222	Reference		Reference		Reference	
≥ 24	56	0.97 (0.62–1.51)	0.880	0.89(0.600–1.33)	0.583	0.97(0.64–1.48)	0.894
*KPS*							
70	3	2.89(0.87–9.65)	0.084	2.07(0.63–6.82)	0.233	2.60(0.78–8.63)	0.119
80	349	0.83(0.53–1.30)	0.414	0.91(0.61–1.37)	0.665	0.90(0.58–1.38)	0.625
90	47	Reference		Reference		Reference	
*Hypertension*							
No	254	Reference		Reference		Reference	
Yes	45	1.38(0.89–2.16)	0.149	1.07(0.71–1.63)	0.744	1.26(0.82–1.93)	0.298
*Diabetes*							
No	290	Reference		Reference		Reference	
Yes	9	0.66(0.21–2.07)	0.477	0.61(0.23–1.63)	0.322	0.57(0.18–1.79)	0.336
*T category*							
1–2	103	Reference		Reference		Reference	
3	143	1.27(0.86–1.90)	0.233	1.19(0.84–1.68)	0.337	1.29(0.89–1.89)	0.182
4	53	1.85(1.15–2.98)	0.011	1.75(1.15–2.66)	0.010	1.81(1.15–2.85)	0.010
*N category*							
0	37	Reference		Reference		Reference	
1	106	1.56(0.84-3.00)	0.152	1.73(0.98–3.05)	0.057	1.47(0.81–2.65)	0.204
2	130	1.56(0.85–2.97)	0.147	1.80(1.03–3.14)	0.038	1.52(0.85–2.72)	0.157
3	26	2.93(1.41–6.08)	0.004	2.91(1.49–5.69)	0.002	2.78(1.40–5.52)	0.003
*GTV, cc*							
≤ 23.27	50	Reference		Reference		Reference	
> 23.27	249	2.58(1.70–3.93)	< 0.001	1.96(1.39–2.76)	< 0.001	2.35(1.59–3.47)	< 0.001
*GTVnd, cc*							
≤ 8.50	210	Reference		Reference		Reference	
> 8.50	89	2.67(1.90–3.80)	< 0.001	2.21(1.62–3.02)	< 0.001	2.65(1.90–3.69)	< 0.001
*Delayed time, days*							
≤ 3	231	Reference		Reference		Reference	
> 3	68	1.48 (1.00-2.20)	0.050	1.29(0.91–1.85)	0.157	1.58(1.10–2.30)	0.015

## Discussion

Esophageal cancer is one of the most common tumors in China [10]. Most patients with locally advanced esophageal carcinoma missed the best time for surgical treatment. Radiation therapy has become important [11, 12]. More interest was aroused in RT sustainability. Evidence was confirmed that RTI had unattractive effects on the prognosis of patients with cancer [13–15]. Cheng X et al [6] found that patients with 5 days of delayed time had lower OS and FFS in nasopharyngeal carcinoma. However, there is rarely research reporting the impact of delayed time on the prognosis of esophageal cancer. Our study discovered that delayed time could cause worse survival outcomes for patients with ESCC.

RTI prolonged the overall treatment time and resulted in dismal survival. Our results confirmed that RTI affects the survival of patients suffering from ESCC. The delayed time was an independent prognostic factor of OS and LRFS. The mortality risk of patients with longer delayed time (> 3 days) was significantly increased (HR = 1.68, 95% CI 1.10–2.55, *p* = 0.016) compared to patients with shorter delayed time (≤ 3 days). Meanwhile, the risk of local recurrence was increased (HR = 1.74, 95% CI 1.18–2.57, *p* = 0.006) for the patients with longer delayed time (> 3 days). Nishimura Y [16] found that the rate of esophageal cancer local control treated with external RT lost 2.3% per day of delay, and 1.4% per day for combined with intraluminal brachytherapy. Yao JJ et al. [17] proposed similar conclusions, The delayed time ≥ 7 days had significant adverse effects on the prognosis of nasopharyngeal carcinoma, and the groups with the longer RTI had poorer survival rates. For the T3-T4 stage, RTI ≥ 5 days led to worse outcomes [18]. It was noticeable that their results (Yao et al.) were based on the assumption starting on Monday, while we used the actual date. It was indicated that the prolonged overall treatment time significantly influenced the survival outcome of patients with ESCC. The mechanism is mainly that the tumor clonogen cells are activated and accelerated repopulation. More additional dose is needed to kill the new cells and maintain the tumor control probability, which is increased linearly with delayed time (the “dog-leg” shape between total dose and overall time) [19, 20]. Otherwise, the tumor cells will be incompletely eliminated.

Many studies have shown that the influence of RTI was negative on PFS. In the study by Xu GZ et al., the 3-year PFS (72.1% vs. 81.9%, *p* = 0.01) was significantly difference between patients with delayed time > 4 days and those with delayed time ≤ 4 days [21]. Hallemeier CL et al. [22] analyzed the association between RTI and outcomes in patients with esophageal cancer using randomized clinical trials of National Cancer Institute–sponsored NRG Oncology (RTOG8501, RTOG0436, RTOG9415). Radiotherapy overall treatment time > 45 days (vs. ≤ 45 days) has a higher risk of disease-free survival (HR = 1.34, 95% CI 1.01–1.77, *p* = 0.04). The delayed time was more applicable to the variable fractionation schedule than the overall treatment time. Unfortunately, Our findings demonstrated that delay time was not an independent prognostic factor for PFS. From the Kaplan‒Meier curves, the delayed time > 3 days (vs. ≤ 3 days) had an insignificant effect on PFS (*p* = 0.15), but the survival outcomes of patients with delayed time > 3 days were slightly different from the patients with delayed time ≤ 3 days. It was necessary to be vigilant about the impact of treatment interruption on the disease-free survival outcome of esophageal cancer.

In addition, the survival curve displayed that the outcome of patients with shorter delayed time (1 to 3 days) was similar to the non-interruptions (0 days), both were better than the longer delayed time (> 3 days). Skladowski et al. submitted a practical result. They explained the relationship between local tumor control and the position of radiotherapy gaps in laryngeal cancer and reported the survival rate of patients with a gap in the middle period was similar to that of patients who had no gap [23]. This was mainly due to the reoxygenation of hypoxic tumor cells. This finding should be given more attention in clinical practice.

In this treatment course, the incidence of RTI was 49.16% (147/299), because of machinery breakdown, public holidays, replan, and patient reason. 57 patients had multiple interruptions, which implied that more than 3 days of prolongation might be one or more interruptions with different reasons or multiple reasons. The characteristics were compared between patients’ reasons and other reasons that resulted in RT interruptions, as shown in Appendix 1, which excluded the multiple interruptions, and it was found that the N stage and volume of positive lymph nodes were different (*p* = 0.007 and 0.042). It should be continuously sought how these reasons work on RTI in the following work. It was difficult to accurately assess which reason is more likely to lead to a delayed time of > 3 days since lacking the acute and late toxicities and only 68 people experienced a delay of > 3 days. However, researchers deduced toxicity was one of the most common reasons for RTI, closely following equipment damages and/or maintenance [24]. Sapienza Lucas G et al. [25] found that one in every 10 patients presented RTI and it was strongly associated with grade 3 and 4 toxicities for locally advanced rectal cancer treated with neoadjuvant chemotherapy. During the treatment, it is necessary to keep a close watch on the toxicity and intervene timely to prevent RTI.

The prognosis of the patient with esophageal cancer is not only related to the treatment protocol, TNM stage, and the factors mentioned, but also concerned with the patient’s psychological state, social status, and compliance [26–28]. We mainly aimed to evaluate the influence of RTI on the prognosis of patients with ESCC receiving chemoradiotherapy. However, the study had some limitations. First, the chemotherapy regimens and RT fractionation were various, the analyses did not rule out confounding factors. Second, the delayed time was not refined as a continuous or discontinuous interruption, and there was no impact of interruption location on survival outcome. Third, the study failed to predict the reason for a delayed time of > 3 days because of lacking the acute and late toxicities. Finally, this study did not conduct stage subgroup analysis. In the future, the sample size will be expanded for subgroup analysis will be conducted for stage heterogeneity.

## Conclusion

There was a significant correlation between RTI and local control of esophageal cancer. The mortality risk and local recurrence risk of patients with longer delayed time (> 3 days) was higher than that of patients with shorter delayed time (≤ 3 days).

### Electronic supplementary material

Below is the link to the electronic supplementary material.

Supplementary Material 1

Supplementary Material 2

## Data Availability

No datasets were generated or analysed during the current study.

## Abbreviations

RT: Radiotherapy
RTI: Radiotherapy interruption
BMI: Body mass index
GTV: Gross tumor volume
GTVnd: Gross tumor volume of the positive lymph nodes
OS: Overall survival
PFS: Progress-free survival
LRFS: Local-regional recurrence-free survival
IMRT: Intensity modulated radiation therapy
VMAT: Volume modulated arc radiotherapy
CTV: Clinical tumor volume of the primary tumor
CTVnd: Clinical tumor volume of the positive lymph nodes
PTV: Planning tumor volumes
OARs: Organs at risk
UICC/AJCC: Union for International Cancer Control/ American Joint Committee on Cancer
RTOG: Radiation therapy oncology group
CW: Clockwise
CCW: Counterclockwise
CCCS: Collapsed cone convolution superposition
HR: Hazard ratio
CIs: Confidence intervals
LINAC: Linear accelerator
ESCC: Esophageal squamous cell carcinoma
