# Supplementary material for: Influence of radiotherapy interruption on esophageal cancer with intensity-modulated radiotherapy: a retrospective study
Source: BMC Cancer. 2024 May 27;24:646. doi: 10.1186/s12885-024-12383-7 (PMC11129380; doi:10.1186/s12885-024-12383-7)
Supplement: Supplementary file 1 — Supplementary Material 1 [file 12885_2024_12383_MOESM1_ESM.docx]

Appendix 1 Comparison of patients’ characteristics between patient’s reason and other reason

| Characteristics | Patients No.(%) | | *p* |
| --- | --- | --- | --- |
|  | Patients’s reason(n=40) | Other reason (n = 50) |  |
| *Gender* |  |  |  |
| Female | 11(27.50%) | 17(34.00%) | 0.647 |
| Male | 29(72.50%) | 33(66.00%) |  |
| *Age, years* |  |  |  |
| ≤ 74 | 38(95.00%) | 43(86.00%) | 0.289 |
| > 74 | 2(5.00%) | 7(14.00%) |  |
| *Drinking* |  |  |  |
| No | 20(50.00%) | 27(54.00%) | 0.832 |
| Yes | 20(50.00%) | 23(46.00%) |  |
| *Smoking* |  |  |  |
| No | 17(42.50%) | 26(52.00%) | 0.402 |
| Yes | 23(57.50%) | 24(48.00%) |  |
| *Diabetes* |  |  |  |
| No | 38(95.00%) | 49(98.00%) | 0.583 |
| Yes | 2(5.00%) | 1(2.00%) |  |
| *Hypertension* |  |  |  |
| No | 30(75.00%) | 42(84.00%) | 0.304 |
| Yes | 10(25.00%) | 8(16.00%) |  |
| *BMI, kg/m^2^* |  |  |  |
| < 18.5 | 1(2.50%) | 4(8.00%) | 0.341 |
| 18.5-24 | 33(82.50%) | 34(68.00%) |  |
| ≥ 24 | 6(15.00%) | 12(24.00%) |  |
| *KPS* |  |  |  |
| 70 | 1(2.50%) | 1(2.00%) | 0.785 |
| 80 | 31(77.50%) | 42(84.00%) |  |
| 90 | 8(20.00%) | 7(14.00%) |  |
| *T category* |  |  |  |
| 1-2 | 13(32.50%) | 19(40.00%) | 0.453 |
| 3 | 18(45.00%) | 25(46.15%) |  |
| 4 | 9(22.50%) | 6(13.85%) |  |
| *N category* |  |  |  |
| 0 | 3(7.50%) | 9(18.00%) | 0.007 |
| 1 | 8(20.00%) | 22(44.00%) |  |
| 2 | 22(55.00%) | 17(34.00%) |  |
| 3 | 7(17.50%) | 2(4.00%) |  |
| *GTV, cc* |  |  |  |
| ≤ 23.27 | 13(32.50%) | 18(36.00%) | 0.825 |
| > 23.27 | 27(67.50%) | 32(64.00%) |  |
| *GTVnd, cc* |  |  |  |
| ≤ 8.50 | 23(57.50%) | 39(78.00%) | 0.042 |
| > 8.50 | 17(42.50%) | 11(22.00%) |  |
