# Supplementary figures and images for: Influence of radiotherapy interruption on esophageal cancer with intensity-modulated radiotherapy: a retrospective study
Source: BMC Cancer. 2024 May 27;24:646. doi: 10.1186/s12885-024-12383-7 (PMC11129380; doi:10.1186/s12885-024-12383-7)

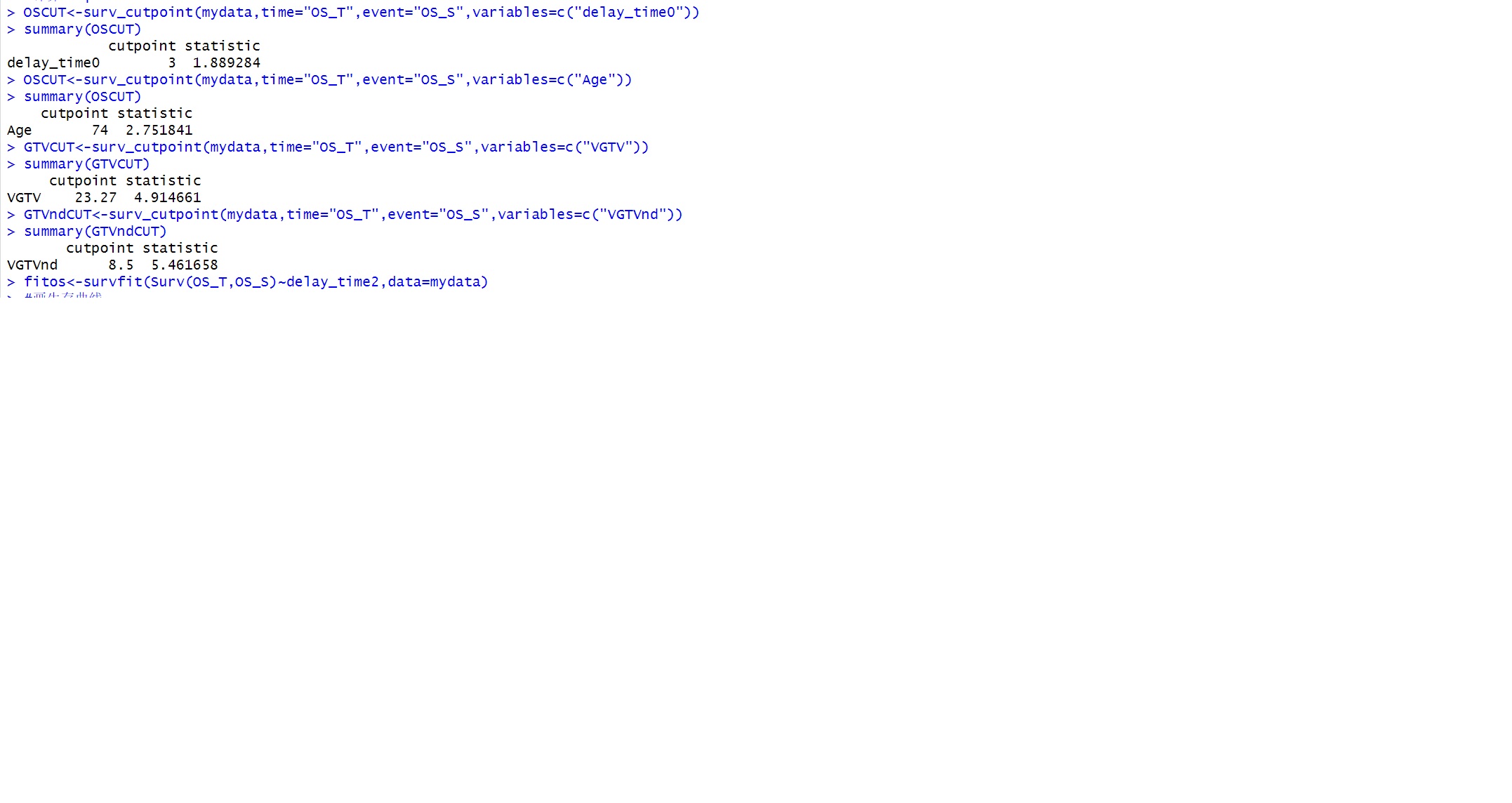

Supplement: Supplementary file 2 — Supplementary Material 2 [file 12885_2024_12383_MOESM2_ESM.jpg]
